# An RNAi-Based Candidate Screen for Modifiers of the CHD1 Chromatin Remodeler and Assembly Factor in *Drosophila melanogaster*

**DOI:** 10.1534/g3.115.021691

**Published:** 2015-11-23

**Authors:** Sharon Kim, Lakshmi Bugga, Eugenie S. Hong, Rebecca Zabinsky, Rebecca G. Edwards, Parimal A. Deodhar, Jennifer A. Armstrong

**Affiliations:** The W.M. Keck Science Department, Claremont McKenna College, Scripps College and Pitzer College, California 91711

**Keywords:** nucleosome, histone dynamics, transcription, modifier screen, chromatin remodeling complex

## Abstract

The conserved chromatin remodeling and assembly factor CHD1 (chromodomains, helicase, DNA-binding domain) is present at active genes where it participates in histone turnover and recycling during transcription. In order to gain a more complete understanding of the mechanism of action of CHD1 during development, we created a novel genetic assay in *Drosophila melanogaster* to evaluate potential functional interactions between CHD1 and other chromatin factors. We found that overexpression of CHD1 results in defects in wing development and utilized this fully penetrant and reliable phenotype to conduct a small-scale RNAi-based candidate screen to identify genes that functionally interact with *chd1 in vivo*. Our results indicate that CHD1 may act in opposition to other remodeling factors, including INO80, and that the recruitment of CHD1 to active genes by RTF1 is conserved in flies.

The complex and dynamic protein-DNA structure that is chromatin regulates DNA accessibility to *trans*acting factors, thereby modulating all DNA-based processes in eukaryotes. Chromatin remodeling factors modulate the position and composition of nucleosomes to regulate transcription and genome organization (Clapier and Cairns 2009; Ryan and Owen-Hughes 2011). The conserved Switch2 (SWI2) family of chromatin remodeling factors (also called SNF2 for sucrose nonfermenting) can be subdivided into 24 subfamilies including SWI2, Imitation SWI (ISWI), INO80 (inositol requiring 80), and CHD1 (chromodomains, helicase, DNA-binding domain) (Flaus *et al.* 2006). The interplay among SWI2 family members (not all of which function as chromatin remodelers) is not fully understood, but multiple chromatin remodeling factors appear to function at a single active gene. For example, five chromatin remodelers (RSC, SWI/SNF, INO80, Isw1, and Chd1) cooperate and function redundantly to remodel the *PHO5* promoter in yeast (Musladin *et al.* 2014).

To date, nine human CHD proteins have been identified, several of which have been linked to various conditions including dermatomyositis, neuroblastoma, and CHARGE syndrome (Marfella and Imbalzano 2007). Loss of human CHD1 is linked to prostate cancer (Huang *et al.* 2011; Liu *et al.* 2011), and mouse Chd1 is required for maintenance of stem cell pluripotency (Gaspar-Maia *et al.* 2009) and early embryogenesis (Suzuki *et al.* 2015). CHD1 is an unusual chromatin remodeling factor in that it is one of the few that possesses *in vitro* nucleosome assembly activity in the presence of a histone chaperone (Lusser *et al.* 2005).

Recent studies have implicated CHD1 as a key factor in chromatin dynamics during transcription *in vivo*. In yeast, Chd1 is required for the maintenance of regular positioning of nucleosomes on genes throughout the genome (Gkikopoulos *et al.* 2011), and Chd1 was recently identified as a factor that is partially responsible for dictating species-specific differences in nucleosome spacing, particularly at the 3′ ends of transcriptionally active genes in *Saccharomyces cerevisiae* and *Kluyveromyces lactis* (Hughes and Rando 2015). In *S. cerevisiae* and mouse embryonic fibroblasts, Chd1 appears to play a dual role at transcriptionally active genes, facilitating turnover of nucleosomes at the 5′ ends of genes while promoting nucleosome retention at the 3′ ends, perhaps via nucleosome recycling during the passage of RNA Polymerase II (Radman-Livaja *et al.* 2012; Skene *et al.* 2014; Smolle *et al.* 2012). In mouse embryonic fibroblasts, Chd1 is important for allowing Pol II to clear the promoter and proceed into the gene body (Skene *et al.* 2014). In metazoans, H3.3 is deposited during transcription-mediated histone replacement (Ahmad and Henikoff 2002a, b). In support of a role in histone turnover, loss of *chd1* (*Chd1*) is associated with a reduction of deposition of the histone variant H3.3 on polytene chromosomes of larval salivary glands in *Drosophila* (Radman-Livaja *et al.* 2012), thus, the basic requirement of Chd1 for nucleosome turnover at active genes appears to be conserved from yeast to flies to mammals.

Many chromatin remodeling factors function in the context of large protein complexes, and subunits of these complexes can play an important role in modulating the function of the ATPase remodeler. However, CHD1 appears to be something of an exception, as a CHD1 protein complex has not been detected (Lusser *et al.* 2005). H3K4me3 peptide affinity purification from HeLa cells identified several factors that may transiently interact with CHD1 including FACT, SPT6, SNF2h (human ISWI), the PAF1 complex, ASH2, and components of the early spliceosome complex (Sims *et al.* 2007). Human CHD1 also binds the NCoR corepressor as well as a histone deacetylase activity (Tai *et al.* 2003), in addition to SSRP1 of the FACT complex and Mediator (Kelley *et al.* 1999; Lin *et al.* 2011). Silkworm CHD1 interacts with HMGA (Papantonis *et al.* 2008), and *Drosophila* CHD1 physically interacts with SSRP of the FACT complex (Kelley *et al.* 1999). In budding yeast, Chd1 and FACT physically interact and genetic studies indicate that they function together in elongation (Simic *et al.* 2003), but Chd1 acts in opposition to FACT at promoters (Biswas *et al.* 2007).

In *Drosophila*, *chd1* is not essential for life, but the gene is critical for male and female fertility as well as wing development (McDaniel *et al.* 2008), and its loss leads to general disruptions in chromosome structure (Bugga *et al.* 2013). In order to identify factors that functionally interact with CHD1 in *Drosophila* and gain insights into its recruitment to active genes, we investigated the colocalization of CHD1 and the H3K4me3 mark on chromosomes, developed a sensitized genetic assay, and conducted a candidate gene screen to fully characterize and validate this new genetic tool. We report evidence for functional relationships between CHD1 and other chromatin remodeling factors including INO80, and identify the transcription elongation factor RTF1 as a protein important for CHD1 recruitment in *Drosophila*.

## Materials and Methods

### Drosophila stocks and crosses

Flies were raised on standard cornmeal-molasses-yeast-agar medium containing Tegosept and propionic acid at 24° unless otherwise indicated. Homozygous *chd1^4^* and *chd1^5^* null mutant larvae (McDaniel *et al.* 2008) were raised at 18°. GAL4-based wing-based assays were performed at 29° unless otherwise indicated to achieve a strong, consistent phenotype. *P[w^+mW.hs^ = GawB]69B* (referred to as *69B-Gal4*), VALIUM RNAi lines (Ni *et al.* 2008), and *PBac[5HPw^+^]lds^A190^/TM3*, *Sb^1^ Ser^1^* were obtained from Bloomington Drosophila Stock Center (http://flystocks.bio.indiana.edu/). Flies carrying the *P[lacW]l(2)SH0566* insertion mapped to the *Rtf1* gene were obtained from the Szeged Drosophila Stock Centre. Lines overexpressing *chd1* and *chd1^KR^*, *w*; *P[w^+^*, *UAS-chd1^+^]126* and *w*; *P[w^+^*, *UAS-chd1^KR^]88*, were constructed from the *chd1* cDNA and were a generous gift from Helen McNeill (Samuel Lunenfeld Research Institute, Mount Sinai Hospital).

To facilitate our candidate screen, we generated a recombinant chromosome carrying both the *UAS-chd1* and *69B-GAL4* transgenes, and maintained the chromosome over a *GAL80*-expressing balancer, thereby preventing the overexpression of *chd1* in the fly stock and reducing the possible accumulation of modifier mutations.

### Generating HA-tagged chd1

To construct a C-terminal fusion protein of CHD1, an in frame 3XHA epitope tag (LGLAYPYDVPDYAGYPYDVPDYAGSYPYDVPDYAAHGGHRST, Drosophila Gateway Vector Project) was incorporated into an 8.5 kb genomic DNA fragment of *chd1* in pCaSpeR2 (McDaniel *et al.* 2008) by overlap PCR (Sambrook and Russell 2001) and cloned into pCR4 TOPO vector (Life Technologies). The resulting construct was subcloned into *pCaSpeR2* carrying the 8.5 kb *chd1* gene (McDaniel *et al.* 2008) using *Bsu*36I and *Avr*II to generate *P[w*^+^, *chd1*^3XHA^*]*. To generate the *chd1^W375L^* mutant, we performed overlap PCR using primers downstream of the *Nhe*I site (+2427) and upstream of the *Mfe*I site (+3595) in conjunction with a mutagenic primer to change the coding sequence from 5′-tgg-3′ to 5′-ttg-3′. The PCR product was cloned into pCR4 using the TOPO cloning kit (Life Technologies) and subcloned into pCaSpeR2-8.5 kb *chd1* 3XHA using *Mfe*I and *Nhe*I to generate *P[w*^+^, *chd1*^3XHA^ *^W375L^]*. Transgenic lines were generated by *P*-element–mediated transformation (Rainbow Transgenics) to generate *P[chd1^W375L^HA*, *w^+^]44-1* and *P[chd1^+^HA*, *w^+^]42-2* fly lines. In both lines, the transgenes are on the X chromosome. To control against errors introduced during PCR amplification or site-directed mutagenesis, all relevant regions were sequenced prior to injection.

### Mounting wings

Right wings of male flies were stored in isopropanol for at least 1 d. Wings were pipetted onto a slide (4–5 wings per slide) and positioned with forceps. Canada Balsam (Sigma-Aldrich) was spread thinly on a coverslip and placed on top of the slide. A weight was placed on coverslips and slides were incubated overnight at 55°. The wings were viewed under a Leica DM 4000 B LED microscope using a 5 × objective.

### Immunostaining of polytene chromosomes

Immunostaining of polytene chromosomes from 3rd instar larvae were performed using several fixation protocols for each antibody (Armstrong *et al.* 2002; Corona *et al.* 2007; Lavrov *et al.* 2004) to ensure that our results were not an artifact from one particular method, a well-documented concern (Johansen *et al.* 2009). Slides with formaldehyde-fixed, squashed chromosomes were blocked and incubated with primary antibodies overnight at 4°. Rabbit anti-CHD1 (McDaniel *et al.* 2008) was used at 1:150 dilution, rabbit anti-RTF1 (a gift from John Lis, Cornell University) was used at 1:50 dilution, rabbit anti-H3K4me3 (Millipore, clone 15-10C-E4) was used at 1:100 dilution, mouse anti-HA (Covance) was used at 1:25 dilution, and mouse IgM anti-Pol IIo^ser2^ (H5) (Covance) was used at 1:50 dilution. Slides were washed and incubated in the appropriate secondary antibodies diluted at 1:200 (Jackson ImmunoResearch) for 1 hr at room temperature, then washed and mounted in Vectashield containing DAPI (Vector Laboratories). Polytene chromosomes were imaged at identical exposure times using an Olympus IX81 or a Leica DM 4000 B LED microscope. Pictures of at least five chromosomes were taken from each slide and 4–12 slides (biological replicates) were prepared for each genotype. Images were processed identically using Adobe Photoshop CS3. To quantify immunofluorescence signals on polytene chromosomes relative to DAPI intensity, we developed a program in Matlab 7.4.0. The code inputs batches of images, each with up to three fluorescent channels, and allows the user to effectively remove nonchromosomal antibody staining by applying a mask that only exposes the polytene chromosomes. To facilitate use, the program has a Java-based graphical user interface. The program can be downloaded from http://faculty.jsd.claremont.edu/jarmstrong/fquant/index.html.

### Coimmunoprecipitation and western blotting

Protein extracts were prepared from 0–12 hr embryos as described (Elfring *et al.* 1998). 500 μg of protein extract was incubated with 25 μl EZ view Red anti-HA Affinity Gel beads (Sigma-Aldrich) in HEGN100 buffer (10 mM HEPES pH 8.0, 1 mM EDTA, 100 mM NaCl, 0.05% Tween-20, 10% glycerol) with protease inhibitors (1 μg/ml each Chymostatin, Leupeptin, Pepstatin A, Aprotinin, and 100 μg/ml PMSF) for 3 hr at 4° on a rotator. Beads were collected at 8200 × *g* for 1 min at 4°, washed in five alternating washes of 10 volumes of HEGN100 and HEGN500 (10 mM HEPES pH 8.0, 1 mM EDTA, 500 mM NaCl, 0.05% Tween-20, 10% glycerol), and samples were eluted in 40 μl 2X SDS sample loading buffer (100 mM Tris-HCl pH 6.8, 4% SDS, 0.2% bromophenol blue, 100 mM DTT, 20% glycerol) by boiling at 95° for 5 min. Western blotting was done following standard procedures with anti-CHD1 at 1:1000 (McDaniel *et al.* 2008) and anti-dRtf1 at 1:3000, with goat anti-rabbit-HRP at 1:10,000 (Bio-Rad), and were detected with Super Signal West Dura (Pierce). Four biological replicates were done.

### Data availability

Fly lines are available upon request.

## Results

### Recruitment of CHD1 to active genes

**Figure 1:**
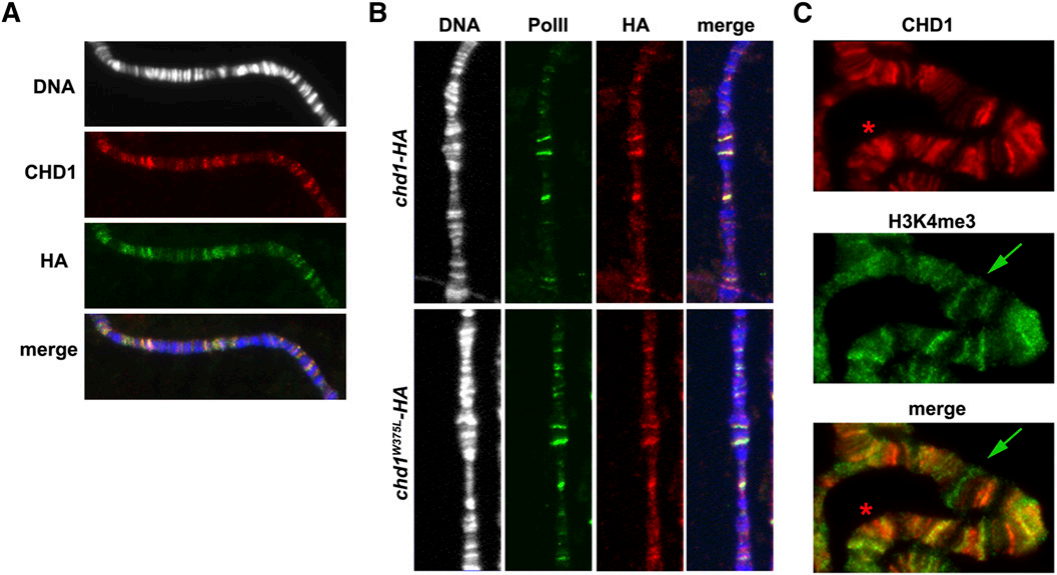
The H3K4me3 mark is not sufficient to recruit CHD1. (A) HA-tagged CHD1 localized to all sites bound by endogenous CHD1. Chromosomes were stained with DAPI (white in top panel, blue in merge) and coimmunostained with anti-CHD1 (red) and anti-HA (green) as described (Lavrov *et al.* 2004). (B) CHD1 localization was not altered in *P[chd1^W375L^HA*, *w^+^]44-1* larvae compared to *P[chd1^+^HA*, *w^+^]42-2* larvae. Chromosomes were labeled with DAPI (white in left panel, blue in merge), anti-Pol IIo^ser2^ (green), and anti-HA (red) as described (Lavrov *et al.* 2004), region 46–53 of chromosome 2R shown for comparison of binding patterns. (C) CHD1 does not completely colocalize with the H3K4me3 histone mark in *P[chd1^+^HA*, *w^+^]42-2*; *chd1^5^ b c sp* larvae. While CHD1 (anti-HA, red) and H3K4me3 (green) patterns were similar (yellow in merge), several H3K4me3 sites lacked CHD1 (green arrow), and CHD1 sites occurred without H3K4me3 (red asterisk). Immunostaining was performed as described (Corona *et al.* 2007).

CHD1 is localized to transcriptionally active genes in flies (Srinivasan *et al.* 2005). However, its mechanism of recruitment is not clear. While the double chromodomains of human CHD1 bind di- and trimethylated H3K4 (Flanagan *et al.* 2005; Sims *et al.* 2005), intact chromodomains were not required for the recruitment of *Drosophila* CHD1 to active chromatin, and *in vitro* binding studies examining the binding of *Drosophila* CHD1 chromodomains to H3 methylated on lysine 4 were inconclusive (Morettini *et al.* 2011). To further characterize the relationship between CHD1 and H3K4me3 on *Drosophila* polytene chromosomes, we performed double immunofluorescence to examine the relative distributions of CHD1 and this active histone mark. Since our antibodies directed against H3K4me3 and CHD1 were raised in rabbits, we used a mouse anti-hemagglutinin antibody to detect a HA-tagged form of CHD1. We cloned the HA sequence onto the 3′ end of a genomic *chd1* transgene (the transgene is –456 to +8019 relative to the *chd1* start site). Expression of this transgene is directed by endogenous *chd1* regulatory sequences to avoid overexpression of the tagged protein. The untagged *chd1* transgene rescued the mutant phenotypes of notched wing margins and male and female sterility (McDaniel *et al.* 2008), as did the *chd1-HA* tagged form (wing vein defects were seen in 0% of *chd1* mutant flies rescued with *chd1-HA*). In flies expressing both the tagged protein and endogenous CHD1, all sites detected by the CHD1 antibody were also recognized by the HA antibody, thus the endogenous CHD1 protein does not bind sites not bound by the CHD1-HA fusion protein (Figure 1A). Furthermore, like endogenous CHD1 (Srinivasan *et al.* 2005), CHD1-HA colocalizes with the elongating form of RNA Polymerase II (Pol IIo^ser2^) (Figure 1B, top panels). No binding was detected in the absence of *HA-chd1* expression (Supporting Information, Figure S1). Thus, we proposed that CHD1-HA behaves as endogenous CHD1 and used CHD1-HA to ask whether H3K4me3 and CHD1 colocalize on polytene chromosomes. While we observed a great deal of colocalization, there are H3K4me3 sites lacking CHD1, and vice versa (Figure 1C), suggesting that trimethylation of H3K4 does not ensure CHD1 binding.

Consistent with previous findings (Morettini *et al.* 2010), we found that a mutation of a conserved tryptophan in the first chromodomain (*chd1^W375L^*) did not alter CHD1 localization to chromosomes (Figure 1B, lower panels). This conserved tryptophan is essential for the binding of human CHD1 chromodomains to methylated H3K4 *in vitro* (Flanagan *et al.* 2005), suggesting that fly CHD1 uses a distinct mechanism to bind to active genes. Furthermore, we found that this conserved residue was dispensable for *chd1* function *in vivo*. Flies that lack *chd1* display notches in wing margins and fertility defects (McDaniel *et al.* 2008). Of 279 *trans*heterozygote *chd1* mutant flies (*chd1^4^ b pr/chd1^5^ b c sp*), 20% displayed notched wing defects, compared to 0% (*n* = 468) of *chd1* mutant flies expressing *chd1^W375L^* (*P[chd1^W375L^HA]*; *chd1^4^ b pr/chd1^5^ b c sp)*. Furthermore, the *chd1^W375L^-HA* transgene fully rescued both male and female sterility. Thus, while there is substantial overlap between CHD1 and H3K4me3 on chromosomes, the CHD1 chromodomains do not appear to be responsible for recruitment of the remodeler to active genes, or for *in vivo* function in flies.

### Development of a sensitized genetic assay to identify chd1 interactors

**Figure 2:**
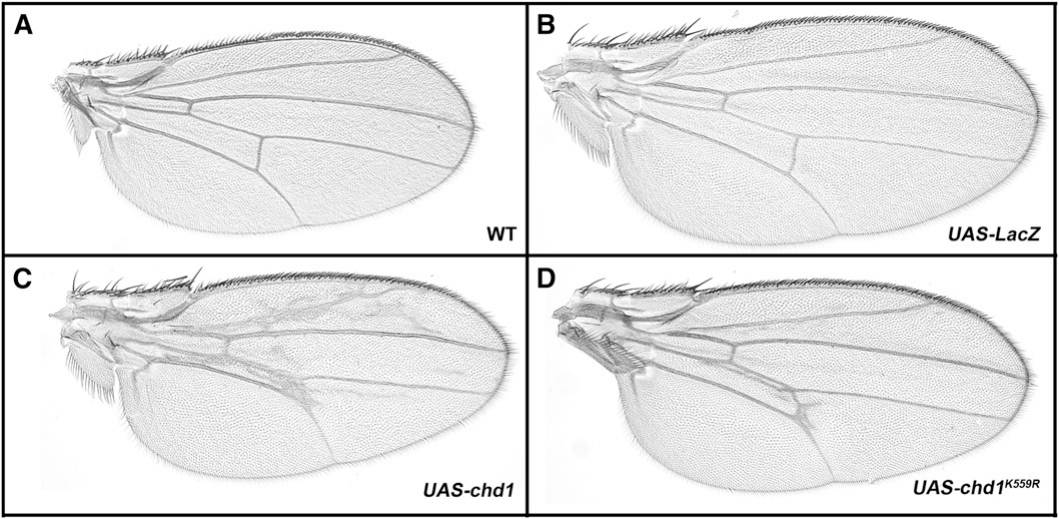
Overexpression of the CHD1 chromatin remodeling factor results in defects in wing development. (A) Wild-type wing from Oregon R fly; (B) wing from *w*; *UAS-lacZ/+*; *69B-Gal4/+* control fly; (C) overexpression of *chd1* resulted in ectopic wing vein formation (*w*; *P[UAS-chd1^+^]/P[69B-Gal4])*; (D) overexpression of *chd1^K559R^* resulted in more modest wing defects (*P[UAS-chd1^K559R^]/P[69B-Gal4]*). All progeny were raised at 24°.

Given that the chromodomains of CHD1 do not appear to be responsible for the recruitment of CHD1 to chromatin, we took a genetic approach to identify factors that may recruit CHD1 to active genes. In our experience, genetic modifier assays have proven valuable in uncovering factors important for the functionality of chromatin remodeling factors in flies (Armstrong *et al.* 2005; Corona *et al.* 2004). With the goal of developing a genetic modifier assay to identify factors important for CHD1, we tested several GAL4 drivers for their ability to generate visible phenotypes upon overexpression of either wild-type *chd1* or *chd1^K559R^* (in which a conserved lysine in the ATPase domain is substituted by an arginine). Expression of either *chd1* or *chd1^K559R^* under control of the *69B-Gal4* driver resulted in visible wing-based phenotypes that were 100% penetrant (Figure 2, C and D, Table S1). No such wing defects were ever detected in wild-type flies (Oregon R) or in control flies overexpressing *LacZ* (Figure 2, A and B). Loss of *chd1* also leads to defects in wing development (McDaniel *et al.* 2008); we therefore chose to develop a wing-based genetic assay, as CHD1 appears critical for the correct formation of this structure.

The mutation of lysine to arginine in the conserved ATPase domain results in dominant-negative alleles in many *Drosophila* chromatin remodeling factors, including Brahma (BRM) and Imitation Switch (ISWI) (Corona *et al.* 2004), presumably because the overabundant ATPase mutants sequester protein subunits away from functional remodelers. In mouse embryonic fibroblasts, either knockdown of Chd1 or expression of Chd1 harboring the corresponding lysine to arginine substitution (K510R) resulted in reduced nucleosome occupancy in the promoter and body of active genes, suggesting that the mutation has the potential to function as a dominant negative (Skene *et al.* 2014). However, *Drosophila* CHD1 has not been identified in a stable protein complex (Lusser *et al.* 2005) and while overexpression of *chd1^K559R^* in salivary glands resulted in polytene chromosomal defects, those defects did not exactly resemble chromosomal phenotypes resulting from loss of *chd1* function (Bugga *et al.* 2013). Given this complexity, we chose to focus on the gain-of-function phenotype resulting from overexpression of wild-type *chd1*.

**Figure 3:**
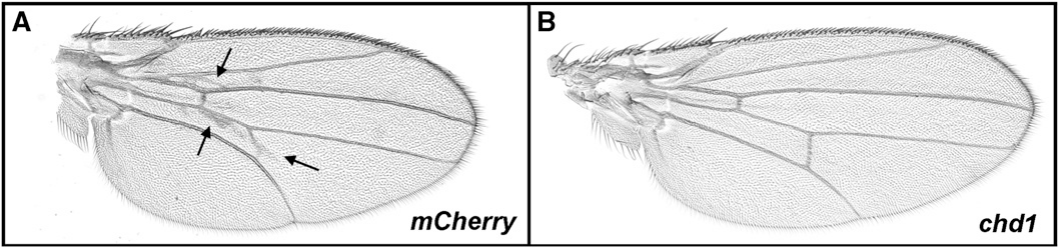
Wing defects resulting from overexpression of *chd1* are suppressed by shRNA directed against *chd1*. (A) Individual overexpressing *chd1* and expressing a shRNA directed against *mCherry* (*w*; *P[UAS-chd1^+^]*, *P[69B-Gal4]/P[VALIUM20-mCherry]attP2*) displayed wing vein defects (arrows). (B) Knockdown of *chd1* by shRNA resulted in nearly complete rescue of wing defects (*P[UAS-RNAi-chd1]/+* ; *P[UAS-chd1]*, *P[69B-Gal4]/+*).

To confirm that the observed defects in wing development were a result of overexpression of *chd1*, we used a VALIUM20-based UAS-driven short hairpin RNA to simultaneously knockdown *chd1* expression with a VALIUM20-based hairpin RNA directed against *mCherry* as a negative control (Ni *et al.* 2011). Wing defects resulting from overexpression of *chd1* were less severe in flies coexpressing the UAS-driven *mCherry* shRNA (compare Figure 3A to Figure 2C), suggesting that GAL4 from the *69B-Gal4* driver may be limiting, as was previously described for the *C96-Gal4* driver (Ni *et al.* 2008). It was thus critical to use flies expressing the *mCherry* hairpin RNA as a baseline control. Expression of *chd1* shRNA resulted in nearly complete suppression of the wing defect phenotype (Figure 3B), confirming that overexpression of *chd1* is responsible for the defects in wing development. Expression of VALIUM20 shRNAs often results in phenotypes resembling null alleles (Ni *et al.* 2011), and immuno-staining of polytene chromosomes confirmed that expression of VALIUM20 *chd1* shRNA resulted in a loss of CHD1 on chromosomes, as well as mild defects in global chromosome structure (Figure S2), as we have previously observed in animals lacking *chd1* (Bugga *et al.* 2013).

### A candidate screen for genes that interact with chd1

**Figure 4:**
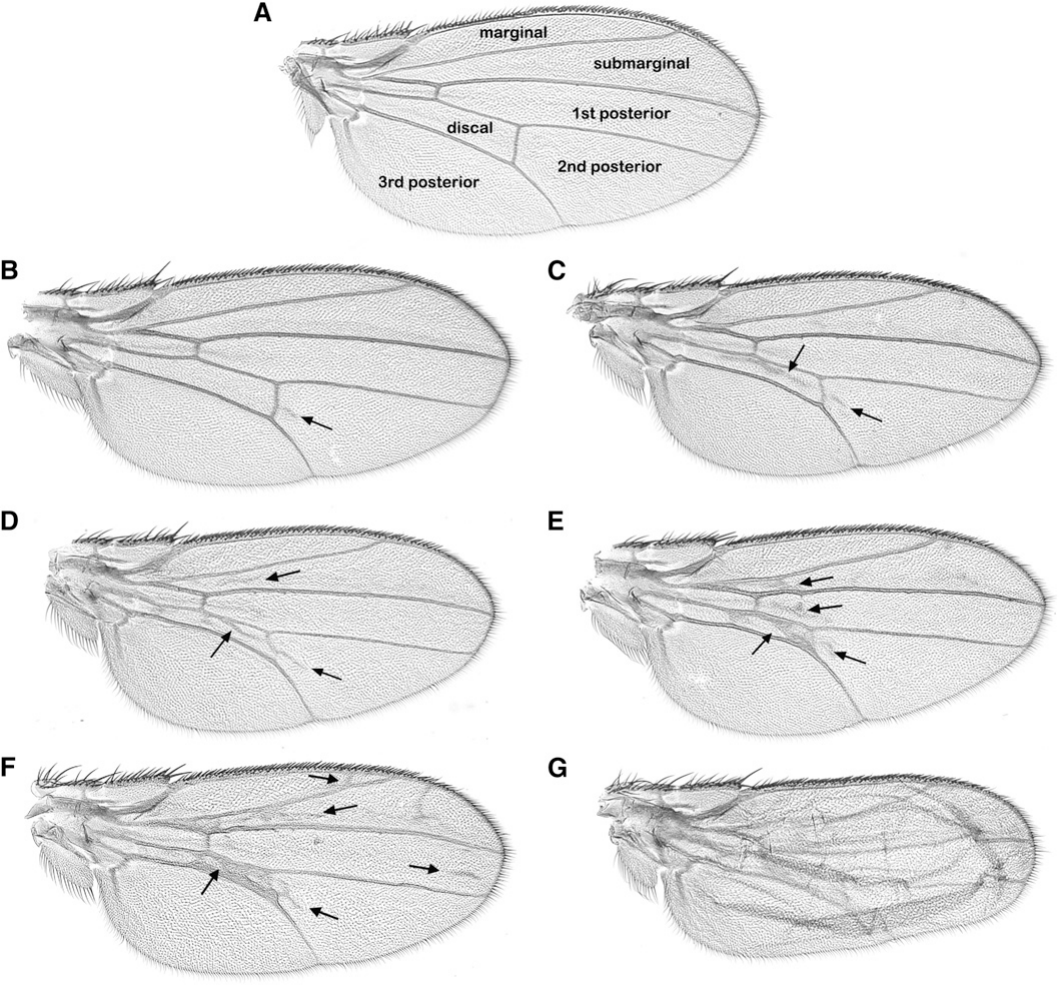
A scoring system for wing defects. Wings were scored for the appearance of ectopic wing veins. (A) A wild-type wing was given a score of 0; (B) a score of 1 was given to wings with ectopic wing veins present in one of the wing cells (arrow); (C–F) a score of 2 through 5 was given to wings with ectoptic wing veins present in two to five of the wing cells (arrows); (G) a score of “B” was given to blistered wings.

One would predict that knockdown of a factor important for CHD1 function would suppress the wing defect phenotype caused by overexpression of *chd1*, while knockdown of a factor that functions antagonistically to CHD1 would enhance wing defects. In order to better evaluate any possible phenotypic changes in this screen, we developed a semiquantitative scoring system. The most common wing defect observed was the appearance of ectopic wing vein structures (Figure 2C and Figure 3A). We therefore scored the appearance of ectopic wing veins in five areas of the wing: the marginal, submarginal, first posterior, discal, and second posterior cells (we never observed ectopic wing veins in the third posterior cell). Wild-type wings received a score of 0 (Figure 4A). A score of 1 was given to a wing if an ectopic wing vein appeared in one of the wing cells, a score of 2 was given if wing veins were apparent in two cells of the wing, etc. (Figure 4, B–F). We occasionally observed wings that appeared blistered, possibly as a result of loss of cell adhesion between the dorsal and the ventral wing surfaces (Figure 4G); these wings were given a score of B and were not included in our subsequent statistical analysis.

**Figure 5:**
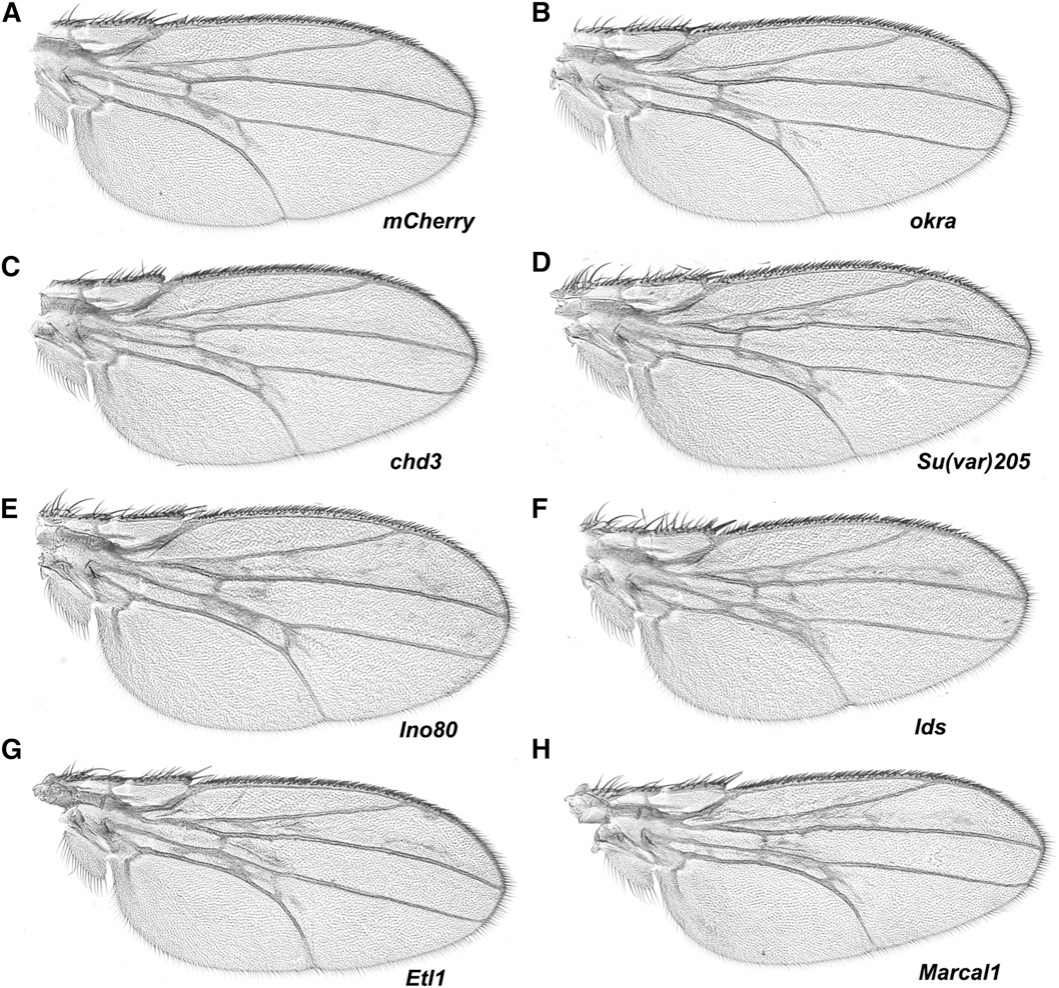
Expression of shRNA directed against *Su(var)205*, *Ino80*, *lds*, *Etl1*, or *Marcal1* enhances wing defects caused by *chd1* overexpression. (A) shRNA-*mCherry* control (*w*; *P[UAS-chd1]*, *P[69B-Gal4]/P[VALIUM20-mCherry]attP2)*. (B) Expression of shRNA directed against *okra* or (C) *chd3* did not modify the *chd1* overexpression wing defects. Expression of shRNA directed against (D) *Su(var)205*, (E) *Ino80*, (F) *lds*, (G) *Etl1*, or (H) *Marcal1* enhanced wing defects as compared to the *mCherry* control. shRNA, small hairpin RNA.

**Figure 6:**
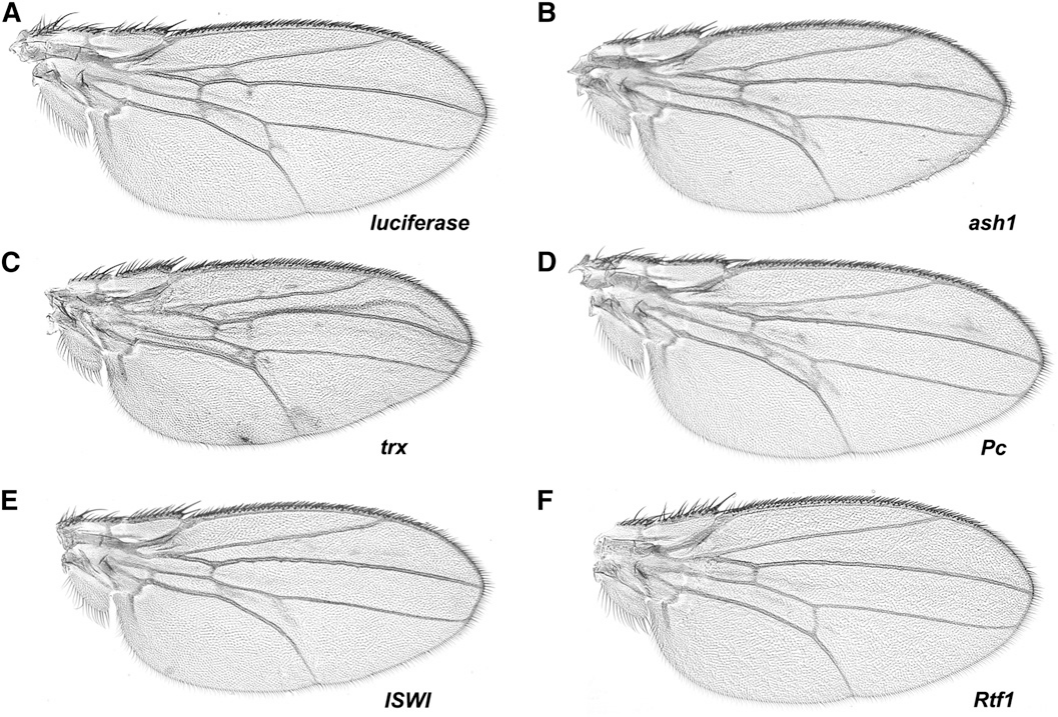
Expression of hairpin RNA directed against *trx* or *Pc* enhances wing defects while hairpin RNA directed against *ISWI* or *Rtf1* suppressed wing defects. (A) RNAi-*Luciferase* control (*w*; *P[UAS-chd1]*, *P[69B-Gal4]/P[VALIUM1-luciferase]*). (B) Hairpin RNA directed against *ash1* did not modify wing defects. Hairpin RNA directed against (C) *trx* or (D) *Pc* enhanced wing defects, while hairpin RNA directed against (E) *ISWI* or (F) *Rtf1* suppressed the wing defect phenotype.

As a candidate gene screen, we selected VALIUM-based RNAi lines from the Transgenic RNAi Project (TRiP) (Ni *et al.* 2008, 2011) that target genes that encode factors important for chromatin dynamics. shRNA directed against *mCherry* was used as a baseline for the VALIUM20 lines and *Luciferase* long hairpin RNA was used as a baseline for the VALIUM1 lines. Knockdown of many of our chosen genes using the *69B-gal4* driver resulted in lethality or near lethality, these targets included *domino*, *XNP*, *Mi-2*, *brahma*, *E(z)*, *Su(var)3-3*, *Su(var)2-10*, *lid*, *Ssrp*, *Stat92E*, *Nipped-B*, *spt4*, and *Nedd8*. This is not surprising as genes encoding proteins important for chromatin structure are often essential. Expression of hairpin RNA targeting *Utx*, *Su(z)12*, *Chd3*, *ash1*, *HP1c*, or *okra* failed to modify the *chd1* wing defects (Figure 5, Figure 6, Table 1, and Table 2). Note that given challenges with viability, the numbers of progeny were at times somewhat low. Hairpin RNA targeting *Bre1* did not result in overt changes in wing veins, but did result in a large percentage of flies with blistered wings (Table 2), suggesting some manner of interaction between *Bre1* and *chd1*. Expression of hairpin RNA targeting *Ino80*, *lodestar* (*lds*), *Etl1*, *Su(var)205*, *Marcal1*, *trithorax* (*trx*), or *Polycomb* (*Pc*) enhanced *chd1*-induced wing defects (Figure 5 and Figure 6), suggesting that the products of these genes may function antagonistically toward CHD1. Expression of hairpin RNA targeting *ISWI* (*Iswi*) suppressed the ectopic wing vein structures and resulted in loss of the posterior cross-vein (Figure 6 and Table 2). As a control, we confirmed that expression of hairpin RNA targeting these candidate genes did not result in wing defects in the absence of overexpression of *chd1* (Figure S3), suggesting that the observed changes in wing phenotypes were due to functional interactions between the candidate factors and CHD1. To confirm that these genetic interactions were not limited to RNAi lines, we asked whether *P* element insertion alleles of two genes (*lds* and *Rtf1*) were able to dominantly modify the *chd1* gain of function phenotype. Indeed, *lds^A190^* enhanced the wing phenotype while *P[lacW]l(2)SH0566*, an insertion mapped to the *Rtf1* gene, suppressed the phenotype (Figure S4).

**Table 1:** Genetic interactions in *chd1* wing-based assay

Hairpin RNA	Wing Score							
	0	1	2	3	4	5	B	*P* Value
*mCherry*	0	1	3	12	1	0	0	
*chd1*	7	21	6	0	0	0	0	<0.001
*Su(var)205*	0	1	3	12	1	0	0	<0.001
*Ino80*	0	0	0	2	7	11	11	<0.001
*lds*	0	0	0	10	6	4	4	<0.001
*Etl1*	0	1	2	14	20	0	0	<0.001
*Marcal1*	0	0	1	7	8	0	0	<0.001
*okra*	0	2	7	7	4	0	0	0.492
*chd3*	0	0	6	15	6	0	0	0.149
*Utx*	0	0	4	6	0	0	1	0.291
*Su(Z)12*	0	0	2	8	0	0	0	0.903

Virgin females of the *69B-GAL4,UAS-chd1*/balancer stock were mated to males of the VALIUM20 RNAi stock of interest. Wings of the appropriate progeny class were scored on a scale from 0–5, as described in Materials and Methods and Figure 3. *P* values were determined using the Mann–Whitney *U*-test, comparing progeny from the experimental cross to progeny from the *mCherry* control crosses.

**Table 2:** Genetic interactions in *chd1* wing-based assay

Hairpin RNA	Wing Score							
	0	1	2	3	4	5	B	*P* Value
*Luciferase*	0	5	20	35	7	0	0	
*trx*	0	0	1	9	2	0	1	0.062
*Pc*	0	0	2	9	14	0	1	<0.001
*ISWI*	0	13	24	4	0	0	0	<0.001
*Rtf1*	6	7	1	1	0	0	0	<0.001
*ash1*	0	2	8	14	6	0	0	0.415
*Bre1*	1	2	6	5	1	0	18	0.087
*Hp1c*	0	0	7	25	5	0	0	0.061

Virgin females of the *69B-GAL4,UAS-chd1*/balancer stock were mated to males of the VALIUM1 RNAi stock of interest. Wings of the appropriate progeny class were scored on a scale from 0 5, as described in *Materials and Methods* and Figure 3. *P* values were determined using the Mann–Whitney *U*-test, comparing progeny from the experimental cross to progeny from the *luciferase* control crosses.

**Figure 7:**
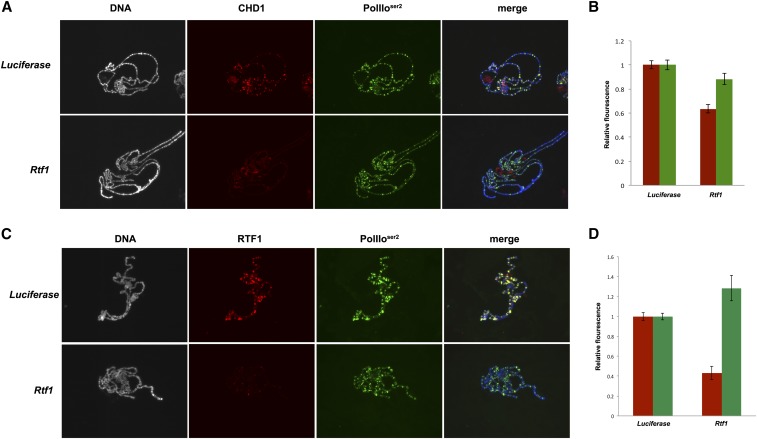
Knockdown of *Rtf1* by RNAi results in decreased levels of CHD1 binding. (A) Chromosomes stained with DAPI (white in left panel, blue in merge) and coimmunostained with anti-Pol IIo^ser2^ (green) and anti-CHD1 (red) from larvae expressing hairpin RNA directed against *Luciferase* (control) or *Rtf1*. (B) Quantification of immunofluorescence of anti-Pol IIo^ser2^/DAPI (green bars) and anti-CHD1/DAPI (red bars) indicates that CHD1 protein levels (relative to DAPI) were reduced by 36% on chromosomes from *Rtf1* knockdown salivary glands (*n* = 10) as compared to expression of *luciferase* hairpin RNA (control, *n* = 11); student’s *t*-test *P* = 3.2 × 10^−7^. Pol IIo^ser2^ levels were not statistically different (*P* = 0.074). (C) Chromosomes stained with DAPI (white in left panel, blue in merge) and immunostained with anti-Pol IIo^ser2^ (green) and anti-RTF1 (red) from larvae expressing hairpin RNA directed against *Luciferase* (control) or *Rtf1*. (D) Quantification of immunofluorescence of anti-Pol IIo^ser2^/DAPI (green bars) and anti-RTF1/DAPI (red bars) indicates that RTF1 protein levels (relative to DAPI) were reduced by 57% on chromosomes from *Rtf1* knockdown salivary glands (*n* = 11) as compared to *Luciferase* RNAi (control, *n* = 15); student’s *t*-test *P* = 6.5 × 10^−8^. Levels of Pol IIo^ser2^ were slightly increased upon knockdown of *Rtf1* (*P* = 0.022). Immunostaining was performed as described (Lavrov *et al.* 2004).

In budding yeast, Rtf1 is a subunit of the transcriptional elongation PAF1 complex and is required for the recruitment of Chd1 to active genes (Simic *et al.* 2003). In *Drosophila*, Rtf1 colocalizes with Paf1 on active genes, but it is not a stable component of the PAF1 complex (Adelman *et al.* 2006). Consistent with a model in which *Drosophila* RTF1 is required for CHD1 binding to active genes, we observed that expression of hairpin RNA targeting *Rtf1* suppressed wing defects resulting from *chd1* overexpression (Figure 6 and Table 2). Furthermore, we observed reduced levels of CHD1 on salivary gland polytene chromosomes derived from salivary glands expressing *Rtf1* hairpin RNA (Figure 7, A and B). Polytene immunostaining confirmed that RTF1 levels were reduced by approximately 60% following knockdown of *Rtf1* by hairpin RNA (Figure 7, C and D). To ask if a physical interaction is responsible for the recruitment, we performed coimmunoprecipitation from embryo extracts, but failed to detect a stable interaction between CHD1 and RTF1 (Figure S5). Consistent with a model in which RTF1 is required for the recruitment of CHD1, but not vice versa, the loss of CHD1 did not result in a reduction in RTF1 levels on polytene chromosomes (Figure S6). Unexpectedly, the levels of RTF1 increased 150% following knockdown of *chd1*, while the global levels of elongating RNA Polymerase II on chromosomes remained unchanged, as we have previously observed (McDaniel *et al.* 2008).

## Discussion

In this study, we describe a new *in vivo* genetic assay to allow the identification of factors that functionally interact with *Drosophila* CHD1. We performed a focused candidate gene screen and identified several genes, including SWI2 family members, as potential CHD1 interaction partners. Given that this is an RNAi-based screen, we took several steps to reduce the possibility of nonspecific effects. First, we used both VALIUM1 and VALIUM20 lines in order to test as many different hairpin RNAs for a candidate gene as possible. However, many of our chosen lines resulted in lethality in both of the RNAi systems, while the use of VALIUM20-based RNAi lines to target several genes (*Rtf1*, *ISWI*, *Pc*, *ash1*, and *trx*) resulted in lethality, most likely because the shRNAs used in those lines lead to more effective knockdown of the target gene than the VALIUM1-based lines (Ni *et al.* 2011). Second, given that long double-stranded RNAs can produce off-target effects, we limited our analysis to lines that were not predicted to produce off-targets, which further restricted our available fly lines. Third, we purposefully limited our screen to VALIUM lines, which allow the use of hairpin RNAs directed against *mCherry* or *Luciferase* as negative controls.

Several pieces of evidence support a functional connection between CHD1 and many of the candidates identified in our study. For example, Chd1 was found to be important for transcription termination of Pol II genes in both *S. cerevisiae* and *Schizosaccharomyces pombe*, with Chd1 functioning redundantly with Isw1 and Isw2 at the *GAL10* gene (Alen *et al.* 2002). It is therefore noteworthy that the ATP-dependent transcription termination factor Lodestar (Xie and Price 1996) was identified as a CHD1 antagonist in this screen, and suggests that CHD1 may play a role in transcription termination in flies.

Our findings suggest that INO80 antagonizes CHD1 activity. A multidimensional protein identification technology (MuDPIT) analysis found that mouse CHD1 may directly or indirectly associate with the remodeler INO80 (Lin *et al.* 2011). The Ino80 chromatin remodeling complex in flies includes Reptin and Pontin (homologs of yeast Rvb1 and Rvb2), Actin, dArp5, and dArp8. This complex associates with the Polycomb group protein Pleiohomeotic (PHO), but is not recruited to PREs by Pho (Klymenko *et al.* 2006). The mammalian homolog of PHO, YY1, interacts with the Ino80 complex in mammalian cells to activate gene expression and facilitate homologous recombination-based repair (Klymenko *et al.* 2006; Vella *et al.* 2012; Wu *et al.* 2007).

Given that CHD1 appears to counteract heterochromatinization in flies (Bugga *et al.* 2013), INO80 may function to promote the spreading of heterochromatic marks, or counteract euchromatic marks at or near active genes. This idea is consistent with a recent study that finds yeast Ino80 functioning at gene boundaries to prevent spreading of the euchromatic mark H3K79me3 by inhibiting Dot1 activity (Xue *et al.* 2015). Additionally, characterization of *Ino80* mutants in flies revealed that INO80 is required for the repression of ecdysone-induced genes during prepupal development (Neuman *et al.* 2014). The idea that chromatin remodeling factors may counterbalance each other to delineate boundaries between active and inactive genomic regions warrants further investigation.

Loss of the SWI2-family member Kismet results in a decrease in the levels of both CHD1 and TRX on polytene chromosomes (Srinivasan *et al.* 2005, 2008), suggesting that the three proteins may function in a pathway. While *trx* VALIUM1 RNAi lines showed reduced viability, we were able to recover progeny that also overexpressed *chd1*. A visual inspection suggested that hairpin RNA targeting *trx* may result in an enhancement of *chd1* wing defects (Figure 6), but the data generated by our scoring system did not reveal a statistically significant change from control wings (Table 2). Analysis of polytene chromosomes derived from animals homozygous for a temperature sensitive *trx* allele (*trx^1^*) raised at the nonpermissive temperature failed to detect changes in CHD1 localization to active genes (Figure S7). Thus, the dependence of chromosome binding by CHD1 and TRX on KIS may occur through independent pathways.

We previously uncovered an unexpected relationship between CHD1 and HP1a, in that overexpression of *chd1* resulted in a decrease in HP1a levels on chromosomes while loss of *chd1* resulted in an increase in HP1a levels (Bugga *et al.* 2013). This antagonistic relationship was also observed in our genetic assay; expression of hairpin RNA targeting *Su(var)205*, which encodes HP1a, resulted in enhancement of the *chd1*-induced wing defects. As the two proteins do not bind to the same sites on chromosomes, the underlying mechanism of a mutual antagonism is unclear.

If a protein functions in concert with CHD1, we would predict that knockdown of that interacting factor would suppress wing defects resulting from overexpression of CHD1. These phenotypes were less common in our screen and were limited to RNAi lines targeting *chd1*, *ISWI*, and *Rtf1*. Yeast Chd1 and Isw1 work together to maintain correct nucleosome spacing across genes starting with the +2 nucleosome (Gkikopoulos *et al.* 2011). In flies, a functional connection between CHD1 and ISWI has not been documented. Indeed, while CHD1 is localized exclusively to active genes (Srinivasan *et al.* 2005), ISWI and RNA Pol II show little overlap on polytene chromosomes from larval salivary glands (Deuring *et al.* 2000). Our findings indicate that *chd1* and *ISWI* may function in concert during wing development.

Yeast Chd1 physically interacts with Rtf1 and is dependent upon Rtf1 for binding to active genes (Simic *et al.* 2003). Rtf1 is an integral subunit of the PAF1 transcription complex in all eukaryotes (reviewed in Jaehning 2010), and human CHD1 was found to associate with the PAF1 complex (Sims *et al.* 2007). Our findings indicate that this relationship is conserved in flies: hairpin RNA directed against *Rtf1* suppresses wing defects caused by overexpression of *chd1* and results in a reduction of CHD1 bound to polytene chromosomes. While we did not find evidence that the two proteins physically interact, both RTF1 and CHD1 are localized to transcriptionally active genes on polytene chromosomes (Adelman *et al.* 2006; Srinivasan *et al.* 2005). In contrast to the yeast proteins, fly RTF1 and PAF1 do not coimmunoprecipitate (Adelman *et al.* 2006). While not a stable subunit of the PAF1 complex, *Drosophila* RTF1 nevertheless colocalizes with PAF1 subunits on polytene chromosomes, is dependent upon PAF1 for binding, and is important for H3K4 methylation patterns at active genes (Adelman *et al.* 2006; Tenney *et al.* 2006). The fly PAF1 complex is not required for the continued binding of elongating RNA Pol II at an induced heat shock gene (Adelman *et al.* 2006), and we observed that RNAi targeting of *Rtf1* did not affect global levels of RNA Pol IIo^ser2^ on polytene chromosomes. RTF1 and CHD1 are not mutually dependent upon each other for binding; instead we observed that loss of CHD1 led to an increase in the levels of R*TF*1 on chromosomes, perhaps as a result of changes in histone dynamics over active genes.

We observed that loss of *chd1* did not alter RTF1 binding to polytene chromosomes, thus CHD1 appears to act downstream of RTF1. Given that human CHD1 binds methylated H3K4 (Flanagan *et al.* 2005; Sims *et al.* 2005), it is possible that the recruitment of fly CHD1 is occurring through a similar mechanism. However, we do not favor this model as we have observed that the H3K4me3 localization pattern is distinct from that of CHD1. Furthermore, *in vitro* studies examining the binding of the CHD1 chromodomains to H3K4 peptides did not reveal that CHD1 preferred methylated peptides (Morettini *et al.* 2011), and we and others observed that intact CHD1 chromodomains are not required for the correct recruitment of CHD1 to its target sites on polytene chromosomes (Morettini *et al.* 2011). Thus, in flies, RTF1 appears to function upstream of both H3K4 methylation and CHD1 binding. These may be independent pathways, as loss of *chd1* does not affect global levels of H3K4 trimethylation (Bugga *et al.* 2013).

Our focused screen has identified several candidate genes that merit further analysis. Expression of hairpin RNAs targeting the SWI2 family members *Ino80*, *lodestar* (*lds*), *Etl1*, *Marcal1*, and *trx* enhance wing defects resulting from overexpression of *chd1*, suggesting that the products of these genes may function antagonistically toward CHD1, while our results suggest that ISWI may function cooperatively with CHD1, as is observed in yeast. Given that Yeast Chd1 appears to function differently at the 5′ and 3′ ends of genes (Radman-Livaja *et al.* 2012; Smolle *et al.* 2012), elucidation of the underlying molecular mechanisms for these genetic observations are likely to reveal exciting aspects of different stages of transcription.

## Supplementary Material

Supporting Information
