# Supplementary material for: An RNAi-Based Candidate Screen for Modifiers of the CHD1 Chromatin Remodeler and Assembly Factor in Drosophila melanogaster
Source: G3 (Bethesda). 2015 Nov 23;6(2):245–54. doi: 10.1534/g3.115.021691 (PMC4751545; doi:10.1534/g3.115.021691)
Supplement: Supporting Information [file supp_6_2_245__index.html]

An RNAi-Based Candidate Screen for Modifiers of the CHD1 Chromatin Remodeler and Assembly Factor in Drosophila melanogaster — Supporting Information 

# An RNAi-Based Candidate Screen for Modifiers of the CHD1 Chromatin Remodeler and Assembly Factor in *Drosophila melanogaster*

## Supporting Information for Kim *et al.*, 2016

**Files in this Data Supplement:**

- Figure S1 - The anti-HA antibody does not show non-specific binding. (.pdf, 5,080 KB)
- Figure S2 - Expression of VALIUM20-based *chd1* sh RNA results in a loss of CHD1 on chromosomes. (.pdf, 6,231 KB)
- Figure S3 - Hairpin RNA directed against candidate genes do not result in wing defects in the absence of *chd1* over-expression. (.pdf, 9,113 KB)
- Figure S4 - Insertion alleles of *Rtf1* and *Ids* dominantly modify the chd1 gain of function wing defects. (.pdf, 2,384 KB)
- Figure S5 - RTF1 does not physically associate with CHD1. (.pdf, 1,436 KB)
- Figure S6 - Loss of CHD1 yields an increase in RTF1 on chromosomes. (.pdf, 7,794 KB)
- Figure S7 - Levels of CHD1 on polytene chromosomes are not affected in *trx* mutant animals. (.pdf, 4,094 KB)
- Table S1 - GAL4 driver survey. (.docx, 48 KB)
