# Supplementary material for: An RNAi-Based Candidate Screen for Modifiers of the CHD1 Chromatin Remodeler and Assembly Factor in Drosophila melanogaster
Source: G3 (Bethesda). 2015 Nov 23;6(2):245–54. doi: 10.1534/g3.115.021691 (PMC4751545; doi:10.1534/g3.115.021691)
Supplement: Supporting Information [file supp_g3.115.021691_FigureS1.pdf]

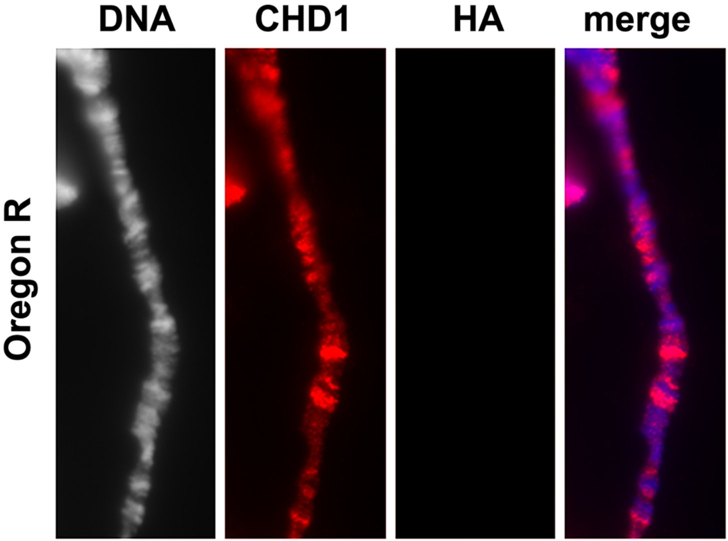

Figure S1. The anti-HA antibody does not show non-specific binding. Oregon R (wild type) chromosomes stained with DAPI (white in left panel, blue in merge) and co-immunostained with anti-CHD1 (red) and anti-HA (green) as described (LAVROV et al. 2004).
