# Supplementary material for: An RNAi-Based Candidate Screen for Modifiers of the CHD1 Chromatin Remodeler and Assembly Factor in Drosophila melanogaster
Source: G3 (Bethesda). 2015 Nov 23;6(2):245–54. doi: 10.1534/g3.115.021691 (PMC4751545; doi:10.1534/g3.115.021691)
Supplement: Supporting Information [file supp_g3.115.021691_FigureS2.pdf]

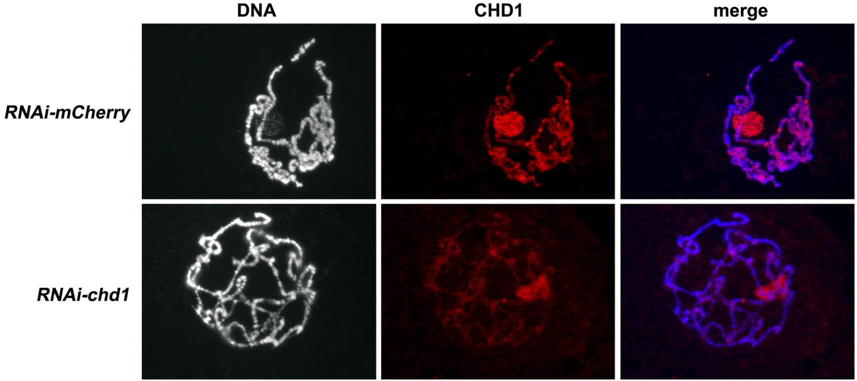

Figure S2. Expression of VALIUM20-based *chd1* shRNA results in a loss of CHD1 on chromosomes. Chromosomes derived from *P[AB1-Gal4]/P[VALIUM20-mCherry]attP2* or *P[AB1-Gal4Gal4]/P[VALIUM20-chd1]attP2* were stained with DAPI (white in left panel, blue in merge) and immuno-stained with anti-CHD1 (red) as described (ARMSTRONG et al. 2002).
