# Supplementary material for: An RNAi-Based Candidate Screen for Modifiers of the CHD1 Chromatin Remodeler and Assembly Factor in Drosophila melanogaster
Source: G3 (Bethesda). 2015 Nov 23;6(2):245–54. doi: 10.1534/g3.115.021691 (PMC4751545; doi:10.1534/g3.115.021691)
Supplement: Supporting Information [file supp_g3.115.021691_FigureS3.pdf]

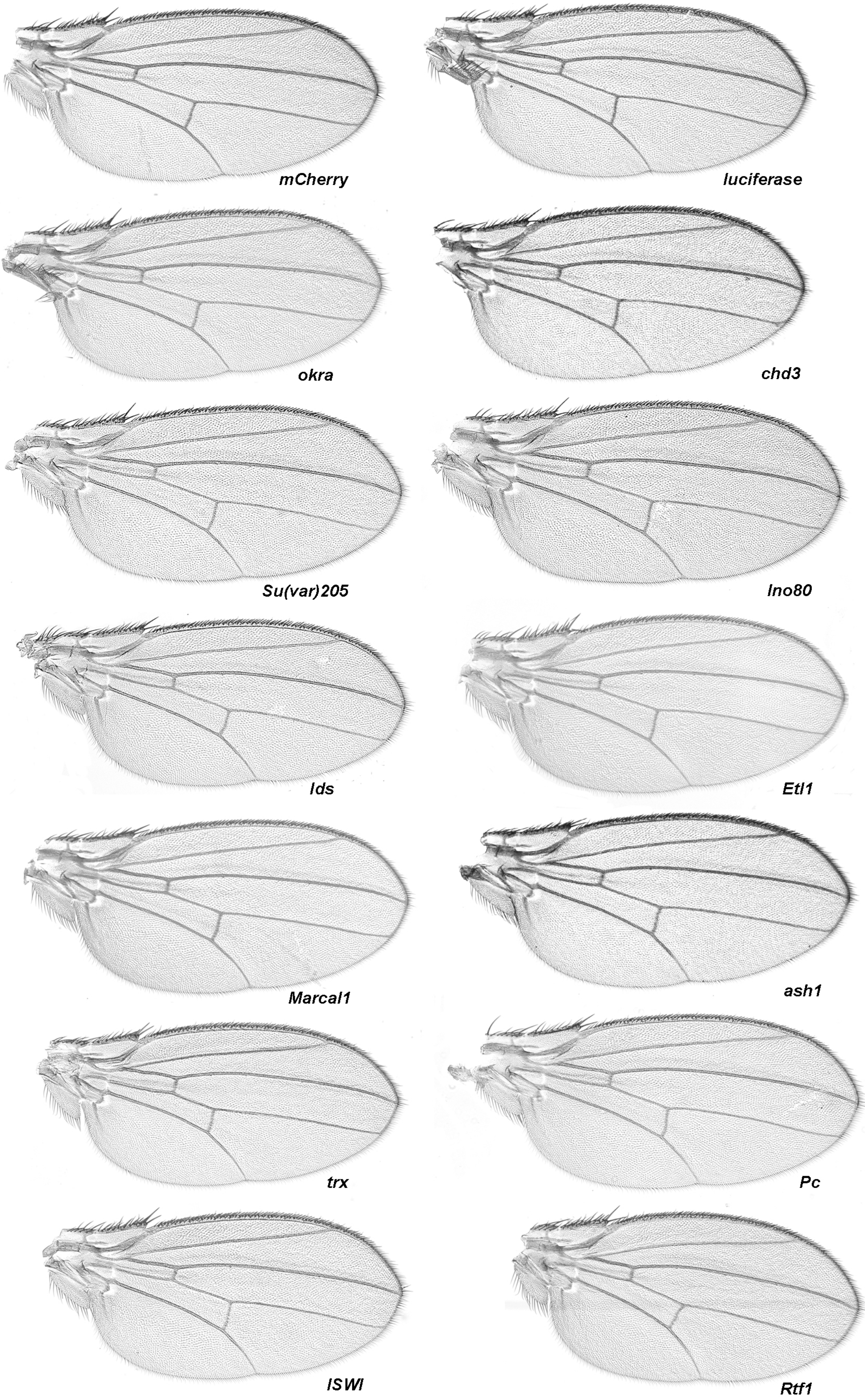

Figure S3. Hairpin RNA directed against candidate genes do not result in wing defects in the absence of *chd1* over-expression. Use of the *69B-Gal4* driver to knockdown *mCherry*, *luciferase*, *okra*, *chd3*, *Su(var)205*, *Ino80*, *lds*, *Etl1*, *Marcal1*, *ash1*, *trx*, *Pc*, *ISWI* or *Rtf1* did not result in defects in wing development.
