# Supplementary material for: An RNAi-Based Candidate Screen for Modifiers of the CHD1 Chromatin Remodeler and Assembly Factor in Drosophila melanogaster
Source: G3 (Bethesda). 2015 Nov 23;6(2):245–54. doi: 10.1534/g3.115.021691 (PMC4751545; doi:10.1534/g3.115.021691)
Supplement: Supporting Information [file supp_g3.115.021691_FigureS4.pdf]

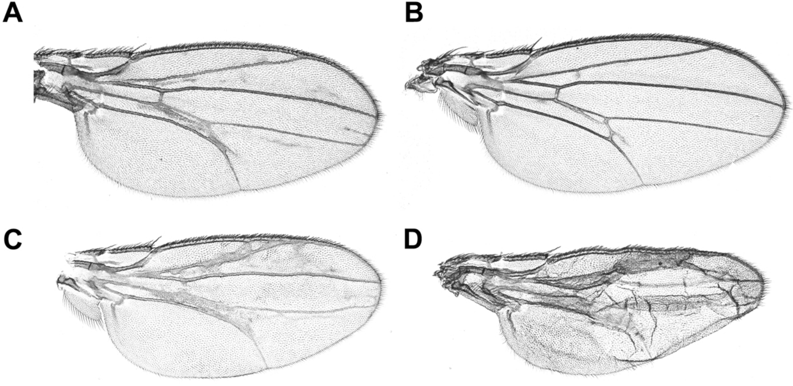

Figure S4. Insertion alleles of *Rtf1* and *Ids* dominantly modify the chd1 gain of function wing defects. (A) Wings from *w; P[UAS-chd1], P[69B-Gal4]/*+, (B) *w; P[lacW]l(2)SH0566/*+; *P[UAS-chd1], P[69B-Gal4]/*+, and (C,D) *w; P[UAS-chd1], P[69B-Gal4]/PBac[5HPw⁺]Ids*^{A190} of which 33% (n=64) were blistered in appearance (D).
