# Supplementary material for: An RNAi-Based Candidate Screen for Modifiers of the CHD1 Chromatin Remodeler and Assembly Factor in Drosophila melanogaster
Source: G3 (Bethesda). 2015 Nov 23;6(2):245–54. doi: 10.1534/g3.115.021691 (PMC4751545; doi:10.1534/g3.115.021691)
Supplement: Supporting Information [file supp_g3.115.021691_FigureS5.pdf]

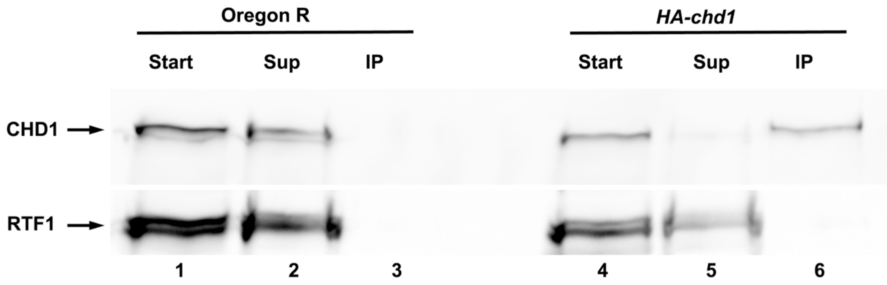

Figure S5. RTF1 does not physically associate with CHD1. Immunoprecipitation of HA-tagged CHD1 from 0-12 hour embryo extracts did not reveal a stable interaction between RTF1 and CHD1 (lane 6). Oregon R, which lacks HA-tagged CHD1, was included as a negative control. Sup (supernatant), IP (immunoprecipitate).
