# Supplementary material for: An RNAi-Based Candidate Screen for Modifiers of the CHD1 Chromatin Remodeler and Assembly Factor in Drosophila melanogaster
Source: G3 (Bethesda). 2015 Nov 23;6(2):245–54. doi: 10.1534/g3.115.021691 (PMC4751545; doi:10.1534/g3.115.021691)
Supplement: Supporting Information [file supp_g3.115.021691_FigureS6.pdf]

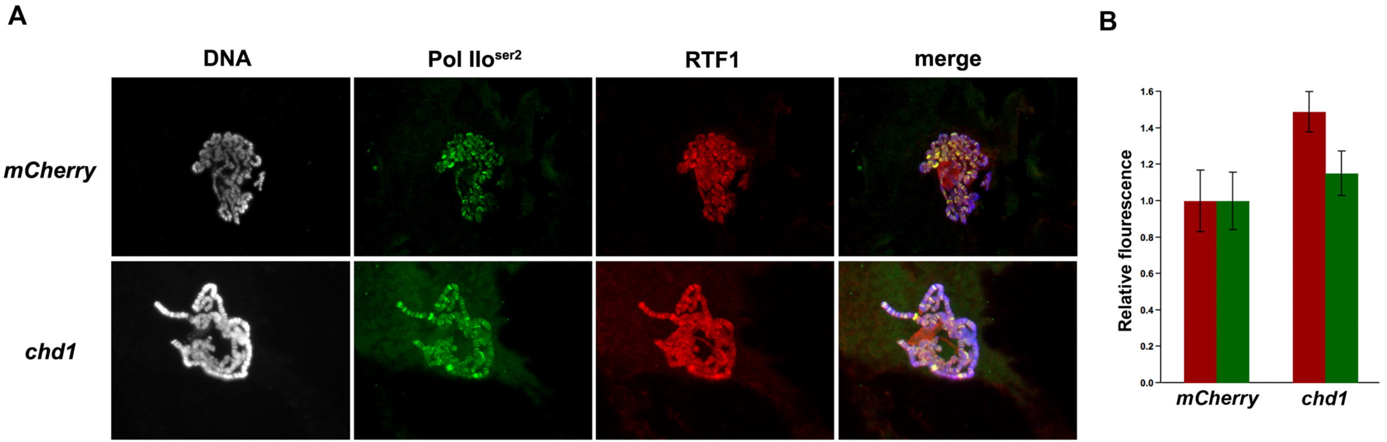

Figure S6. Loss of CHD1 yields an increase in RTF1 on chromosomes. (A) Chromosomes derived from *P[AB1-Gal4]/P[VALIUM20-mCherry]attP2)* or *P[AB1-Gal4]/P[VALIUM20-chd1]attP2* were stained with DAPI (white in left panel, blue in merge) and co-immunostained with anti-RTF1 (red) and anti-Pol IIoser2 (green) as described (LAVROV et al. 2004). (B) Quantification of immunofluorescence of anti-Pol IIoser2/DAPI (green bars) and anti-RTF1/DAPI (red bars) indicates that loss of CHD1 resulted in a 150% increase in RTF1 levels (n= 19) compared to *mCherry* RNAi control (n=17); student's t-test p=0.026. Levels of Pol IIoser2 were unchanged following knockdown of *chd1* (p=0.325).
