# Supplementary material for: An RNAi-Based Candidate Screen for Modifiers of the CHD1 Chromatin Remodeler and Assembly Factor in Drosophila melanogaster
Source: G3 (Bethesda). 2015 Nov 23;6(2):245–54. doi: 10.1534/g3.115.021691 (PMC4751545; doi:10.1534/g3.115.021691)
Supplement: Supporting Information [file supp_g3.115.021691_FigureS7.pdf]

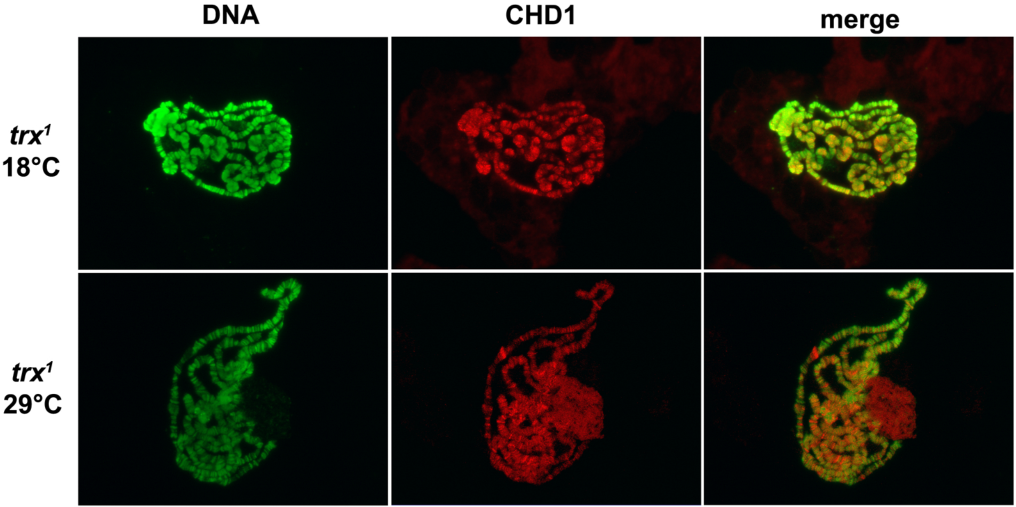

Figure S7. Levels of CHD1 on polytene chromosomes are not affected in *trx* mutant animals. Chromosomes derived from *trx* mutants raised at 18°C (permissive temperature) and 29°C (non-permissive temperature) were immunostained with anti-CHD1 (red), DNA is stained with DAPI (green). Quantification revealed that CHD1 levels were unchanged (p=0.438, n=9).
