# Supplementary material for: An RNAi-Based Candidate Screen for Modifiers of the CHD1 Chromatin Remodeler and Assembly Factor in Drosophila melanogaster
Source: G3 (Bethesda). 2015 Nov 23;6(2):245–54. doi: 10.1534/g3.115.021691 (PMC4751545; doi:10.1534/g3.115.021691)
Supplement: Supporting Information [file supp_g3.115.021691_TableS1.docx]

**Table S1**

**GAL4 driver survey**

| **GAL4 driver** | ***UAS-chd1*** | ***UAS-chd1^KR^*** |
| --- | --- | --- |
| *P[w^+mW.hs^=GawB]69B* | extra wing vein structures | extra wing vein structures |
| *P[w^+mW.hs^=en2.4-Gal4]e22c* | no phenotype | no phenotype |
| *P[w^+mC^=GAL4-Hsp70.PB2* | semi-lethal with wing defects | lethal |
| *P[w^+mC^=Act5C-GAL4]17bFO1* | lethal | semi-lethal |
| *P[w^+mC^=tubP-GAL4]LL7* | lethal | lethal |
| *P[w^+mC^=longGMR-GAL4]2* | no phenotype | no phenotype |
| *P[w^+m*^=GAL4]A9* | malformed wing veins | thickened cross veins |
| *P[w^+m*^=GAL4-vg.M]2* | no phenotype | no phenotype |
